# Factors associated with MRI success in children cooled for neonatal encephalopathy and controls

**DOI:** 10.1038/s41390-022-02180-y

**Published:** 2022-07-29

**Authors:** Kathryn Woodward, Arthur P. C. Spencer, Sally Jary, Ela Chakkarapani

**Affiliations:** 1 Translational Health Sciences, Bristol Medical School, University of Bristol, Bristol, UK; 2 Clinical Research and Imaging Centre, University of Bristol, Bristol, UK

## Abstract

**Objective:**

To investigate if an association exists between motion artefacts on brain MRI and comprehension, co-ordination, or hyperactivity scores in children aged 6–8 years, cooled for neonatal encephalopathy (cases) and controls.

**Methods:**

Case children (*n* = 50) without cerebral palsy were matched with 43 controls for age, sex, and socioeconomic status. Children underwent T1-weighted (T1w), diffusion-weighted image (DWI) brain MRI and cognitive, behavioural, and motor skills assessment. Stepwise multivariable logistic regression assessed associations between unsuccessful MRI and comprehension (including Weschler Intelligence Scale for Children (WISC-IV) verbal comprehension, working memory, processing speed and full-scale IQ), co-ordination (including Movement Assessment Battery for Children (MABC-2) balance, manual dexterity, aiming and catching, and total scores) and hyperactivity (including Strengths and Difficulties Questionnaire (SDQ) hyperactivity and total difficulties scores).

**Results:**

Cases had lower odds of completing both T1w and DWIs (OR: 0.31, 95% CI 0.11–0.89). After adjusting for case-status and sex, lower MABC-2 balance score predicted unsuccessful T1w MRI (OR: 0.81, 95% CI 0.67–0.97, *p* = 0.022). Processing speed was negatively correlated with relative motion on DWI (*r* = −0.25, *p* = 0.026) and SDQ total difficulties score was lower for children with successful MRIs (*p* = 0.049).

**Conclusions:**

Motion artefacts on brain MRI in early school-age children are related to the developmental profile.

**Impact:**

- Children who had moderate/severe neonatal encephalopathy are less likely to have successful MRI scans than matched controls.
- Motion artefact on MRI is associated with lower MABC-2 balance scores in both children who received therapeutic hypothermia for neonatal encephalopathy and matched controls, after controlling for case-status and sex.
- Exclusion of children with motion artefacts on brain MRI can introduce sampling bias, which impacts the utility of neuroimaging to understand the brain–behaviour relationship in children with functional impairments.

## Introduction

Motion artefacts in magnetic resonance imaging (MRI) occur frequently in practice and are problematic as this can impair the clinical and research utility of scans. Motion artefacts occur in response to the voluntary or involuntary movement of subjects during scanning and can appear as blurring or ghosting,^1^ distorting the shape, size,^2^ and diffusion properties of the tissues imaged.^3,4^

In studies comparing subjects with and without neurological or developmental disorder, inclusion of noisy brain MRI data can lead to false findings.^4^ On the other hand, exclusion of data from subjects with motion artefact can result in sampling bias, as the excluded subjects may have poorer outcome scores and may show more alterations in brain structure or tissue properties. This loss of data can therefore impact the utility of neuroimaging to understand the brain–behaviour relationship in subjects with functional impairments.

Subject motion during MRI is especially prevalent with children,^5–8^ who may find it difficult to stay still in a noisy and confined space for the time required to obtain the MRI image in the scanner. Motion artefact in MRI is more common in younger than older children,^6,8,9^ and may also be related to increased anxiety,^10^ and/or movement or attention impairments^9^ associated with conditions such as attention deficit hyperactivity disorder (ADHD),^11^ autism spectrum disorder (ASD) and epilepsy.^9^ Furthermore, children with unsuccessful MRI have shown lower scores on assessments of cognitive and language skills,^12^ unsuccessful MRI in children with ASD and ADHD has been associated with sensorimotor atypicalities,^8^ and scores quantifying impulsivity in children correlated with head motion during MRI.^13^ However, currently, there is no existing data assessing factors associated with MRI success in early school-age children previously cooled for neonatal encephalopathy (NE) and matched controls. We therefore examined this in a cohort of early school-age children without cerebral palsy (CP), previously cooled for moderate/severe NE and in matched controls who underwent MRI as part of the CoolMRI study.^14^ Our objective was to examine associations between unsuccessful brain MRI scans and comprehension, co-ordination and hyperactivity scores. We hypothesised that children with unsuccessful MRI due to motion artefacts have poorer comprehension and co-ordination skills, and are more hyperactive, than children who had successful MRI without motion artefacts.

## Materials and methods

Participants were recruited through the CoolMRI study, which enrolled 50 case children who received therapeutic hypothermia as a neuroprotective intervention for NE, and 43 control children matched for age, sex, and socioeconomic status. Case children were recruited from the population-based cohort of infants born at >35 weeks gestation and cooled for moderate to severe NE between October 2007 and November 2012 at St Michael’s NICU, Bristol, based on regional protocol.^15^ Children who had NE plus additional diagnoses or who were diagnosed with CP at 2 years of age were excluded. Children in the control group were born at >35 weeks gestation and had no known significant medical condition or perinatal asphyxia and were matched at the group level with cases for age, sex and socioeconomic status as determined by the index of multiple deprivation. Index of multiple deprivation was calculated using the postcode where the child was living at early school-age and was based on a weighted combination of seven domains of deprivation including income, employment, education, housing, health, disability and crime (1 most deprived; 10 least deprived) as defined by the UK Government.^16^ Children with MRI incompatible implants were excluded from the study. Children were between the ages of 6 and 8 years during enrolment in the study between October 2015 and August 2019. The South-West – Frenchay Research Ethics Committee approved this study (REC ID: 15/SW/0148), and written informed consent was obtained from the children’s parents and assent obtained from the children.

### MRI image acquisition

T1-weighted (T1w) and diffusion-weighted images (DWI) were acquired using a Siemens 3T Magnetom Skyra MRI scanner at the Clinical Research and Imaging Centre in Bristol, UK.

To prepare children for MRI scanning, families were provided with a purpose-made video to watch prior to attending the imaging facility which demonstrated the MRI procedures. This video was viewed with participants and families again on the day of the MRI, when each step of the MRI process was explained in a child-friendly way including auditory experience of the different sounds made by the MRI scanner. Children were also given the option to familiarise themselves with lying in the scanner using an in-house custom-made mock MRI. During scanning, all children watched a video of their choice, wore soft silicone ear plugs, and had padding around their heads to help keep their heads still. Sequences were acquired in the same order for each participant, with the T1-weighted scan performed first followed by the DWI scan, and MRI scans took around 30 min in total.

The T1w anatomical scan was acquired with the magnetisation-prepared rapid acquisition gradient echo sequence using the following parameters: echo time (TE) 2.19 ms; inversion time (TI) 800 ms; repetition time (TR) 1500 ms; flip angle 9°; field of view (FoV) 234 × 250 mm; 176 slices; 1.0 mm isotropic voxels. DWI data were acquired with a multi-band echo-planar imaging sequence, using the following parameters: TE 70 ms; TR 3150 ms; FoV 192 × 192 mm; 60 slices; 2.0 mm isotropic voxels, flip angle 90°, phase encoding in the anterior-posterior direction, in-plane acceleration factor = 2 (GRAPPA),^17^ through-plane multi-band factor = 2.^18–20^ For the purpose of data averaging and eddy-current distortion correction, two sets of DWI were acquired with *b* = 1000 s mm^−2^ in 60 diffusion directions, as well as eight interspersed *b* = 0 images, with one data set acquired with positive phase encoding steps, then repeated with negative steps (so-called, “blip-up, blip-down”), giving a total of 136 images.

### Classification of successful and unsuccessful MRI scans

T1 images were visually assessed by two assessors who were blind to the case–control status of the participant. Those with moderate to severe movement artefacts, such as blurring, or ghosting were classified as unsuccessful due to motion artefact. DWI image quality was quantified using the EddyQC tool^21^ from the FMRIB Software Library (http://fsl.fmrib.ox.ac.uk).^22^ This provides measures of the amount of movement (*x*, *y* and *z* translation and rotation, total absolute motion, total relative motion) and distortion (*x*, *y* and *z* eddy currents, total susceptibility-induced distortion) present in the data. Metrics were normalised, and then the root-mean-square across all metrics was calculated, giving a single score that increases monotonically with the amount of movement and distortion. DWIs were classified as unsuccessful if their score was more than one standard deviation above the mean of all participants. We also determined whether children had at least one (≥1) unsuccessful MRI scan, classified as either an unsuccessful T1w or DWI or two unsuccessful scans (T1w and DWI), compared to children who had two successful MRI scans (T1w and DWI, i.e., a full successful MRI battery). Supplementary Fig. 1 provides examples of successful and unsuccessful T1w and DWIs. For correlation analyses, the absolute motion and relative motion were also obtained from EddyQC. Absolute motion indicates how far the subject moved their head from the initial position and is calculated as the displacement of each image in the DWI sequence from the first image in the sequence, averaged over all 136 images. Relative motion indicates how much the subject moved their head from one image to the next and is calculated as the displacement of each image from the preceding image in the sequence, averaged over all 136 images.

### Neurodevelopmental assessment

Cognitive skills were assessed using the Wechsler Intelligence Scale for Children (WISC-IV).^23^ The WISC-IV includes 10 subtests to assess four domains: verbal comprehension, perceptual reasoning, working memory, and processing speed. Raw scores for each subtest were converted into scaled scores, and scaled scores were summed for each domain and converted into domain composite scores (mean 100, standard deviation 15) and a full-scale IQ score.

Motor abilities were assessed using the Movement Assessment Battery for Children (MABC-2).^24^ The MABC-2 includes eight tasks to assess three domains: manual dexterity, aiming and catching, and balance. Raw scores for each task were converted into item standard scores (mean 10, standard deviation 3), and item standard scores were summed to give component standard scores (mean 10, standard deviation 3) for each of the three domains, and an overall score (MABC-2 total score).

Emotional and behavioural development of the children were assessed using the Strengths and Difficulties Questionnaire (SDQ)^25^ completed by parents, which has composite scores for emotional symptoms, conduct problems, hyperactivity/inattention, peer relationship problems and prosocial behaviour, total difficulties, and in addition, an impact score.

### Statistical analyses

We used “*N*−1” *χ*^2^ test to compare the proportions of case and control children with and without motion artefact on MRI. The distribution of continuous variables was assessed using the Shapiro–Wilk test. The association between developmental outcomes and unsuccessful MRI scans due to artefact was analysed using stepwise multivariable binary logistic regression (forward Wald) after adjusting for case–control status and sex. Given the association between the cognitive and motor and behavioural skills,^26^ we constructed separate multivariable binary logistic regression models for cognitive, motor and behavioural skills. The outcome variable included unsuccessful T1w (yes/no); unsuccessful DWI (yes/no); one or more unsuccessful sequence (yes/no). The independent variables for comprehension (cognitive) skills included WISC-IV verbal comprehension, working memory, processing speed and full-scale IQ; for co-ordination (motor) skills included MABC-2 manual dexterity, aiming and catching, balance, and MABC-2 total score and for hyperactivity (behavioural skills) included SDQ hyperactivity and total difficulties score. To examine the association between these independent variables and two DWI movement parameters (absolute motion and relative motion) measured using EddyQC, we applied bivariate correlations. Further analyses also compared these independent variables between children who did not go into the MRI scanner, children with at least one unsuccessful MRI sequence (T1w or DWI), and children with a successful MRI battery using the Kruskal–Wallis test or one-way ANOVA, depending on the distributions of the variables. Post hoc pairwise analysis was assessed using two-sample Wilcoxon tests. IBM SPSS statistics version 21 was used for statistical analyses.

## Results

**Fig. 1:**
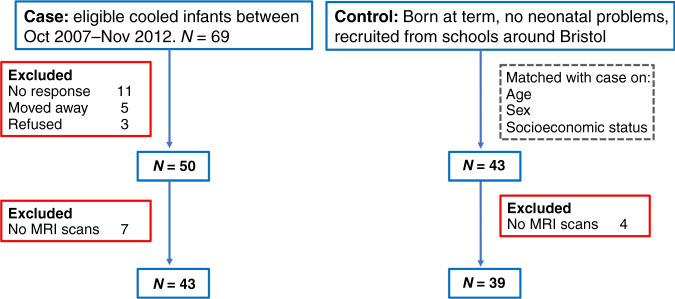
Participant flowchart. Flow chart showing the number of case and control children recruited.

Participant flowchart is shown in Fig. 1. Of the 69 eligible case children, 50 children were included, and 43 control children matched for age, sex, and socioeconomic status were included (Supplementary Table 1). Of 93 children in the cohort, 11 children (7 cases) did not undergo brain MRI scans, leaving 82/93 (88%) children with MRI scans, which included 43/50 (86%) case children and 39/43 (91%) control children. A further four case children had incomplete DWI sequences (4/43). There were no significant differences in the proportion of case and control children who underwent MRI scans (*p* = 0.511). Of 82 children who underwent MRI, cases had a significantly lower odds of completing a full successful MRI battery (T1w and DWI) compared to controls (27/60 (45%) vs 33/60 (55%); OR 0.31, 95% CI: 0.11–0.89, *p* = 0.03) There were no significant differences between cases and controls with and without successful T1w (OR: 0.33, 95% CI: 0.1–1.15) or DWI (OR: 0.38, 95% CI: 0.1–1.60) scans (Table 1).

**Table 1:** Number and proportion of case and control children with successful and unsuccessful scans.

MRI sequences	Unsuccessful	Successful	*p* value
T1w and DWI, *n* (%)	22 (23.83)	60 (76.17)	0.03
Cases/Controls, *n*	16/6	27/33	
T1w scan, *n* (%)	15 (18.29)	67 (81.71)	0.08
Cases/Controls, *n*	11/4	32/35	
DWI scan, *n* (%)	10 (12.82)	68 (87.20)	0.2
Cases/Controls, *n*	7/3	32/36	

### Associations between developmental outcomes and unsuccessful MRI

A summary of developmental outcome scores is presented in Supplementary Table 2. Significant variables in individual stepwise regression models for comprehension, co-ordination and hyperactivity are presented in Tables 2–4, respectively. After controlling for case–control status and sex, only unsuccessful T1w images were negatively associated with MABC-2 balance scores, whereby for each 1-point increase in the balance standard score, the odds of an unsuccessful T1w image decreases by 19% (odds ratio: 0.81, 95% CI 0.67–0.97, *p* = 0.022; Table 3). All other associations between MRI success and developmental outcomes (independent variables) were not significant. A significant association was also found between the case-status control variable and children with at least one unsuccessful scan, whereby case children were 3.24 times more likely to have at least one unsuccessful scan compared to control children (odds ratio: 3.24, 95% CI 1.11–9.42, *p* = 0.031; Tables 2–4), further supporting the association between case-status and odds of completing a full successful MRI battery reported above.

**Table 2:** Stepwise multivariate binary logistic regression examining the association between MRI scan success and comprehension skills, controlling for case–control status and sex.

Outcome variable	Variables allowed	Independent variables	Coefficient, *B*	Odds ratio, Exp(*B*)	95% CI Exp(*B*)	*p* value
Unsuccessful T1w	Control^a^ and comprehension skills^b^ variables	Constant	−2.19	0.11		<0.001*
		Case-status	1.10	3.01	0.87, 10.44	0.082
		Female	0.04	1.04	0.33, 3.27	0.946
Unsuccessful DWI	Control^a^ and comprehension skills^b^ variables	Constant	−2.22	0.11		0.001*
		Case-status	0.93	2.54	0.60, 10.72	0.205
		Female	−0.53	0.59	0.15, 2.32	0.452
≥1 unsuccessful sequence	Control^a^ and comprehension skills^b^ variables	Constant	−1.62	0.20		0.002*
		Case-status	1.18	3.24	1.11, 9.42	0.031*
		Female	−0.15	0.86	0.31, 2.36	0.769

*p < 0.05.

^a^Control variables include case-status and sex (female).

^b^Comprehension skills variables include WISC-IV verbal comprehension, working memory, processing speed and full-scale IQ.

**Table 3:** Stepwise multivariate binary logistic regression examining the association between MRI scan success and co-ordination skills, controlling for case–control status and sex.

Outcome variable	Variables allowed	Independent variables	Coefficient, *B*	Odds ratio, Exp(*B*)	95% CI Exp(*B*)	*p* value
Unsuccessful T1w	Control^a^ and co-ordination skills^b^ variables	Constant	0.13	1.15		0.911
		Case-status	0.88	2.42	0.66, 8.84	0.182
		Female	−0.10	0.91	0.27, 3.03	0.875
		MABC-2 balance	−0.22	0.81	0.67, 0.97	0.022*
Unsuccessful DWI	Control^a^ and co-ordination skills^b^ variables	Constant	−2.22	0.11		0.001*
		Case-status	0.93	2.54	0.60, 10.72	0.205
		Female	−0.53	0.59	0.15, 2.32	0.452
≥1 unsuccessful sequence	Control^a^ and co-ordination skills^b^ variables	Constant	−1.62	0.20		0.002*
		Case-status	1.18	3.24	1.11, 9.42	0.031*
		Female	−0.15	0.86	0.31, 2.36	0.769

*p < 0.05.

^a^Control variables include case-status and sex (female).

^b^Co-ordination skills variables include MABC-2 manual dexterity, aiming and catching, balance and total MABC-2.

**Table 4:** Stepwise multivariate binary logistic regression examining the association between MRI scan success and hyperactivity, after controlling for case–control status and sex.

Dependant variable	Variables allowed	Independent variables	Coefficient, *B*	Odds ratio, Exp(*B*)	95% CI Exp(*B*)	*p* value
Unsuccessful T1w	Control^a^ and hyperactivity^b^ variables	Constant	−2.19	0.11		<0.001*
		Case-status	1.10	3.01	0.87, 10.44	0.082
		Female	0.04	1.04	0.33, 3.27	0.946
Unsuccessful DWI	Control^a^ and hyperactivity^b^ variables	Constant	−2.22	0.11		0.001*
		Case-status	0.93	2.54	0.60, 10.72	0.205
		Female	−0.53	0.59	0.15, 2.32	0.452
≥1 unsuccessful sequence	Control^a^ and hyperactivity^b^ variables	Constant	−1.62	0.20		0.002*
		Case-status	1.18	3.24	1.11, 9.42	0.031*
		Female	−0.15	0.86	0.31, 2.36	0.769

*p < 0.05.

^a^Control variables include case-status and sex (female).

^b^Hyperactivity variables include SDQ hyperactivity and total difficulties scores.

### Associations between DWI motion metrics and developmental outcomes

Bivariate correlations revealed a significant negative correlation between WISC-IV processing speed and average relative motion (*r* = −0.25, *p* = 0.026). There were no other significant correlations between motion metrics measured during DWI and the developmental outcome measures (*p* value range 0.071–0.993).

### Differences in outcome scores between children who did not go in the scanner, and those who had unsuccessful and successful MRI scans

Developmental outcome measures were compared between (1) children who did not go in the scanner, and (2) children with and (3) without a successful MRI battery (unsuccessful T1w or DWI). This revealed a significant main effect of the group on children’s SDQ total difficulties scores (*p* = 0.047). Post hoc analyses revealed that children with successful MRI scans had significantly lower SDQ total difficulties scores (median (IQR), 6 (3,11)) than children with at least one unsuccessful MRI (median (IQR), 10 (6,13); *p* = 0.049) (Supplementary Fig. 2). There were no significant differences in SDQ total difficulties scores between children who did not go in the scanner (median (IQR), 12 (5,16)) and children with successful scans (*p* = 0.068) or at least one unsuccessful scan (*p* = 0.619). There were no other significant main effects of the group when comparing developmental outcomes between children who did not go in the scanner, and children with and without motion artefact on at least one MRI sequence (*p* value range 0.072–0.969). There were also no significant differences in developmental outcomes between children who underwent MRI compared to those who did not (*p* value range 0.084–0.918).

## Discussion

Among early school-age children who underwent brain MRI, there was a greater proportion of cases (children without CP cooled for moderate to severe NE) than control children with an unsuccessful MRI battery due to artefact. After adjusting for case–control status and sex, the only significant association between MRI success and the developmental outcome was on T1w scan success and MABC-2 balance scores. For every unit increase in balance score, the odds of an unsuccessful T1w scan decreased by 19%. There was also a significant negative correlation between WISC-IV processing speed and average relative head motion during DWI acquisition, and children with an unsuccessful MRI battery had higher SDQ total difficulties scores than children with a successful MRI battery. These findings highlight associations between aspects of children’s developmental profile and unsuccessful MRI due to motion artefact.

### Children cooled for NE are more likely to have unsuccessful brain MRI scans due to motion artefact than matched controls

A greater proportion of children who were cooled for NE (cases) had unsuccessful brain MRI scans at early school age due to motion artefact than control children who were matched for age, sex, and socioeconomic status. Overall, 54% of cases (27/50) and 77% of controls (33/43) completed successful MRI scans (both T1w and DWI) without motion artefact. These findings are similar to those seen in children aged 7–9 years with ADHD (39–47%), ASD (56%) and matched controls (83–91%) who completed an fMRI battery without excessive head motion.^9^ The lower success rates of MRI scanning for children with ADHD is thought to be related to symptoms of hyperactivity, impulsivity, and inattention,^27^ or to co-morbid anxiety.^28^ For children with ASD, lower success rates may also relate to impaired motor performance,^29^ as well as difficulties with communication and comprehension, and increased sensitivity to noise.^30^ Our cohort differs from the participants in these studies in that none of the children in our cohort had ASD or ADHD diagnoses and were also younger. It is not yet known whether some may go on to fulfil diagnostic criteria for ADHD or ASD. Nevertheless, high risk of unsuccessful scans in children previously cooled for NE could be related to their developmental profile.

### Developmental profile of children with and without motion artefact on brain MRI

Both cases and control children who had unsuccessful T1w MRI scans had significantly lower scores on measures of balance than children with successful scans. Furthermore, the association between motion artefact on MRI and balance skills was independent of case–control status, suggesting this association was not due to a larger proportion of case than control children with unsuccessful scans. Reduced balance and co-ordination skills may impact a child’s ability to maintain stability of their head position during MRI scanning, as balance involves the ability to maintain a controlled body position during both static (still) and dynamic (movement) activities.^31^ Simhal et al suggest further research into the role of “soft neurological signs” (NSS) in predicting MRI success is needed.^8^ NSS include poor co-ordination, speed or accuracy of limb or axial movements, including those required to keep balance, and these signs are thought to be markers of brain immaturity. They have been identified during clinical examination of healthy young children but resolve with age.^32^ However, emerging literature suggests the persistence of NSS across a wide variety of neurodevelopmental and biological disorders in children. The ability to accurately execute motor tasks of the MABC-2 requires the successful integration of both physical and neuro-cognitive factors and sub-optimal MABC-2 scores may indicate a need for later school-age cognitive evaluation.^26^

Children with lower processing speed scores made larger movements of their heads during DWI acquisition. Processing speed has been shown to be a significant predictor of verbal comprehension in typically developing children,^33^ and so this finding highlights an association between head motion during scanning and factors relating to verbal and cognitive performance in children. Previous research has shown that children with ASD who had lower verbal and cognitive performance scores showed significantly greater head motion during scanning and reduced neural connectivity on motion-free fMRI volumes compared to children with ASD who had higher verbal and cognitive performance scores.^34^ Therefore, this shows that imaging clinical groups with lower MRI success rates is important to gain valuable insights into brain–behaviour relationships.

Children with an unsuccessful MRI battery also had higher SDQ total difficulties scores than children with a successful MRI battery. Previous research has shown that ability to lie still within a confined and noisy MRI scanner is associated with anxiety in paediatric groups,^35,36^ and children with a history of NE have greater behavioural difficulties, including anxiety, than control children.^37^ Previous research has shown that compliance with MRI procedures is related to a child’s attentional or impulsivity problems.^13,38^ However, no significant differences were found in SDQ hyperactivity scores between children with successful and unsuccessful MRI scans. This suggests that emotional and behavioural factors such as anxiety, but not factors related to hyperactivity, such as inattention or impulsivity, may contribute to excessive movement during scanning in this cohort.

### Children with unsuccessful scans differed across MRI modality

This research implemented multimodal MRI (T1w and DWI) scans and applied different motion artefact detection methods to assess the quality of research data obtained from each modality. Excluding participants with incomplete DWIs, these artefact detection methods identified different groups of children with unsuccessful T1w (12/78) or DWI scans (10/78), where only 4 children had unsuccessful images for both MRI modalities. The T1w scan was performed first, lasted around 3 min, and utilised a visual inspection method to detect motion artefacts. DWIs were performed second, and involved a longer sequence with multiple volumetric scans, and an automated tool for DWI data quality assessment. The findings reported here showed children with unsuccessful T1w scans had significantly lower scores for balance, whereas children with unsuccessful DWIs did not. This difference may relate to the order in which the scans were completed, or due to differences in the sensitivity of the MRI modalities to motion artefact or differences in the methods used to classify MRI success. Children who have greater difficulties with balance and motor skills may be more likely to move in the scanner at the outset of MRI procedures, but after an adjustment or acclimatisation period, these children may remain sufficiently still. On the other hand, the T1w scan may be more susceptible to motion artefact than the DWI, as the T1w scan involves a single volumetric image acquired across the entire 3-min sequence, as opposed to 136 brief (<3s) volumetric scans that can be re-aligned to adjust for movement. The total number of children with an unsuccessful T1w image or unsuccessful DWI was similar, suggesting motion artefact impacts research data quality at comparable rates across these MRI modalities. However, due to inherent differences in the signal acquisition and artefact detection and correction procedures, the severity or sensitivity of motion artefacts cannot be adequately compared across modalities.

### Limitations and strengths

This study combined a cohort of early school-age children without CP previously cooled for NE and matched controls, which could potentially limit the generalisation of our findings. The logistic regression analyses applied to our data controlled for the case–control status of the children, and so the relationship between MRI success and balance skills was independent of case-status. However, further research would be needed to examine the generalisation of this finding to other clinical paediatric groups. Another factor which can limit the generalisation of our findings is the small sample of 82 children. Research which examined sufficient sample sizes for logistic regression, suggests that a sample size of 500 participants provides a close approximation of regression parameters for a target population.^39^

One of the strengths of this research is that the CoolMRI study employed MRI familiarisation protocols to reassure children about the MRI procedure and address the impact of children’s anxiety on successfully completing the MRI. For example, this study utilised a pre-MRI orientation video, which can help prepare children to know what to expect before arriving at the MRI facility.^40^ Furthermore, this study allowed children to view movies during scanning, which is also shown to be an effective tool to help reduce children’s anxiety during scanning.^41^ Children recruited for this study were also given the option to lie in a mock scanner, and previous research has also shown that use of mock scanners can help prepare children for MRI procedures.^42,43^ However, not all children opted to lie in the mock scanner before the MRI in this study.

### Future research

As unsuccessful MRI due to subject motion is especially prevalent with children, future studies may benefit from giving children (particularly cases) additional/extended experience in the mock or real MRI scanner or may have greater success after inviting children to a second scanning visit. In the current study, the parents of children with unsuccessful scans were offered the option of a return visit at a later date, but none of the children opted to attend. Previous research has shown that progressive MRI familiarisation protocols, which involve systematically exposing participants to closer approximations of the real MRI scan (e.g., setting, noise), can improve the quality and success rates of MRI scans in young children.^40^

The use of mock MRI stimulations that provide real-time feedback of head motion has also been shown to improve the success rates of MRI scans in young children^40,44^ For example, the use of simple visual cues during mock scanning, such as displaying stimuli on screen when there is a significant amount of motion, can help with showing children how still they need to be during the MRI scan.^44^ Alternatively, MR-based methods can also be employed, which use navigation data collected during MR acquisition to retrospectively correct for motion.^45–49^ For example, this could include recording additional navigator echoes during acquisition to retrospectively measure in-plane translation and rotational motion.^50^

## Conclusions

We demonstrate that early school-age children without CP who were cooled for moderate to severe NE have lower brain MRI success rates in terms of scan quality than control children matched for age, sex, and socioeconomic status. After controlling for case–control status and sex, a significant negative association was found between unsuccessful brain T1w MRI due to motion artefact and MABC-2 balance scores, whereby for every unit increase in the balance component standard score, the odds of an unsuccessful T1w scan decreased by 19%. In neuroimaging studies, exclusion of children with motion artefacts on brain MRI can therefore introduce sampling bias and impact the utility of neuroimaging to understand the brain–behaviour relationship in children with functional impairments. It is therefore important for MRI research to use methodologies that reduce or correct head motion during MRI scanning.

## Supplementary information

Supplementary information

Motion artefact paper supp data

## Data Availability

All data generated or analysed during this study are included in this published article and its supplementary information files.
