# Supplementary material for: Factors associated with MRI success in children cooled for Neonatal Encephalopathy and controls
Source: Pediatr Res. Author manuscript; Available in PMC 2023 Mar 27. (PMC10033414; doi:10.1038/s41390-022-02180-y)
Supplement: Supplementary Material [file EMS146227-supplement-Supplementary_Material.pdf]

Supplementary table 1. Participant demographics for children who underwent MRI scans.

	**Cases (n=43)**	**Controls (n=39)**	***P* value**
Age, median (range)	7.0 (6.0-7.9)	7.1 (6.1-7.9)	0.941
Sex, male/female	21/22	17/22	0.634
Index of Multiple Deprivation, median (range)	7 (1-10)	7 (2-10)	0.407

Supplementary table 2. Summary of developmental outcome scores for children with and without successful MRI scans.

**Developmental outcome**	**Unsuccessful/Successful MRI, mean (SD)**	
**Comprehension Skills**		
WISC-IV Verbal comprehension	*Unsuccessful/successful T1w:*<br>*Unsuccessful/successful DWI:*<br>*≥1 unsuccessful/2 successful scans:*	97.27 (9.16)/ 104.18 (11.80)<br>100.50 (11.81)/ 103.41 (11.81)<br>99.00 (10.81)/ 104.35 (11.67)
WISC-IV Working memory	*Unsuccessful/successful T1w:*<br>*Unsuccessful/successful DWI:*<br>*≥1 unsuccessful/2 successful scans:*	95.40 (11.12)/ 99.85 (13.85)<br>91.10 (13.96)/ 100.28 (13.33)<br>94.82 (11.75)/ 100.58 (13.77)
WISC-IV Processing Speed	*Unsuccessful/successful T1w:*<br>*Unsuccessful/successful DWI:*<br>*≥1 unsuccessful/2 successful scans:*	100.80 (16.57)/ 102.93 (15.48)<br>101.00 (12.96)/ 102.81 (16.21)<br>101.91 (15.15)/ 102.77 (15.89)
WISC-IV Full-Scale IQ	*Unsuccessful/successful T1w:*<br>*Unsuccessful/successful DWI:*<br>*≥1 unsuccessful/2 successful scans:*	97.20 (10.78)/ 102.54 (14.42)<br>99.30 (13.23)/ 102.10 (14.31)<br>98.64 (12.06)/ 102.63 (14.49)
**Co-ordination skills**		
MABC-2 Manual Dexterity	*Unsuccessful/successful T1w:*<br>*Unsuccessful/successful DWI:*<br>*≥1 unsuccessful/2 successful scans:*	8.73 (2.37)/ 10.40 (3.74)<br>10.40 (2.76)/ 10.01 (3.78)<br>9.77 (2.54)/ 10.22 (3.90)
MABC-2 Aiming and Catching	*Unsuccessful/successful T1w:*<br>*Unsuccessful/successful DWI:*<br>*≥1 unsuccessful/2 successful scans:*	9.07 (3.47)/ 9.82 (3.08)<br>9.30 (2.73)/ 9.68 (3.14)<br>9.27 (3.15)/ 9.83 (3.16)
MABC-2 Balance	*Unsuccessful/successful T1w:*<br>*Unsuccessful/successful DWI:*<br>*≥1 unsuccessful/2 successful scans:*	8.40 (3.09)/ 11.07 (3.43)<br>10.80 (4.26)/ 10.63 (3.49)<br>9.41 (3.50)/ 11.02 (3.44)
MABC-2 Total score	*Unsuccessful/successful T1w:*<br>*Unsuccessful/successful DWI:*<br>*≥1 unsuccessful/2 successful scans:*	8.40 (2.77)/ 10.57 (3.62)<br>10.20 (3.43)/ 10.10 (3.68)<br>9.36 (3.02)/ 10.43 (3.73)
**Hyperactivity**		

SDQ Hyperactivity	*Unsuccessful/successful T1w:*<br>*Unsuccessful/successful DWI:*<br>*≥1 unsuccessful/2 successful scans:*	2.53 (2.20)/ 2.57 (2.30)<br>3.20 (2.57)/ 2.43 (2.28)<br>2.95 (2.36)/ 2.42 (2.24)
SDQ Total Difficulties	*Unsuccessful/successful T1w:*<br>*Unsuccessful/successful DWI:*<br>*≥1 unsuccessful/2 successful scans:*	9.40 (4.69)/ 7.99 (5.56)<br>9.40 (6.41)/ 7.88 (5.32)<br>9.91 (5.26)/ 7.63 (5.38)

Supplementary figure 1. Example of successful and unsuccessful T1w images and DWIs. (A) shows an example of a successful T1w image, and (B-C) show examples of unsuccessful T1w images due to blurring or ghosting. (D) shows an example of a successful DWI, and (E-F) show examples of unsuccessful DWIs.

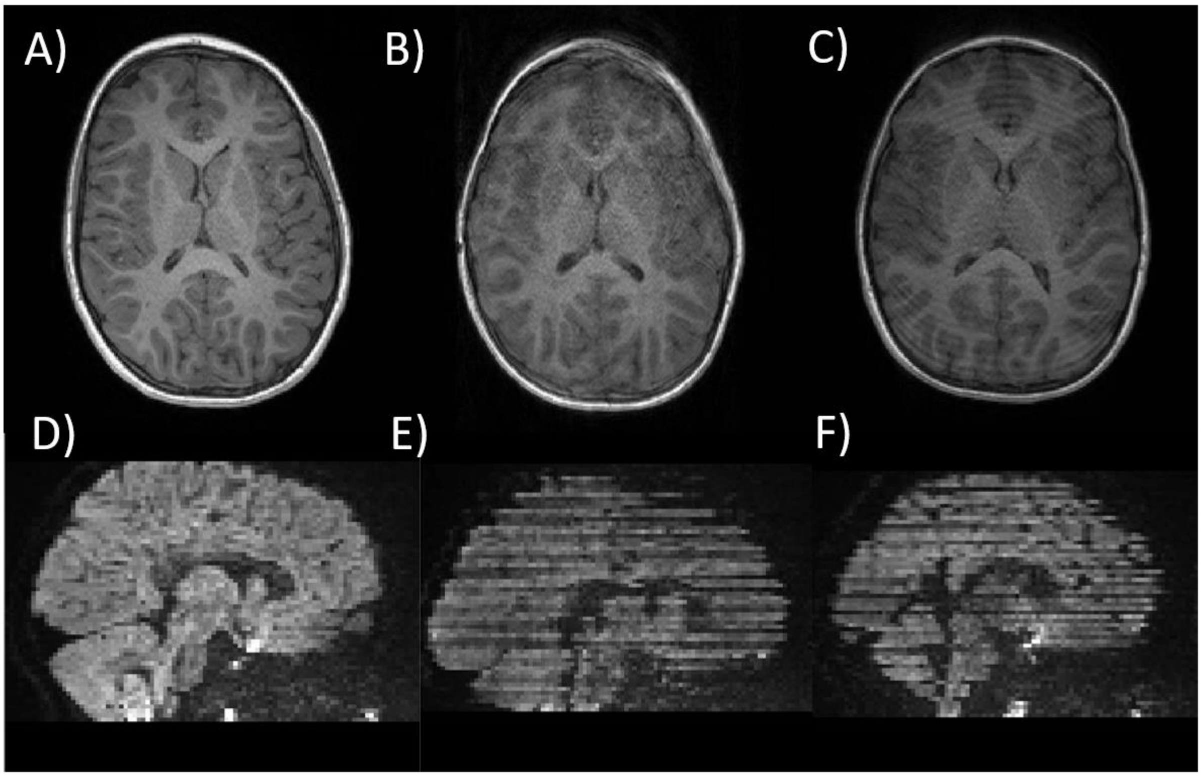

Supplementary figure 2. Comparison of SDQ total difficulties scores between children who did not go in the scanner (n=11), children with at least one unsuccessful MRI (T1w or DWI) (n=22) and children with successful MRIs (n=60).

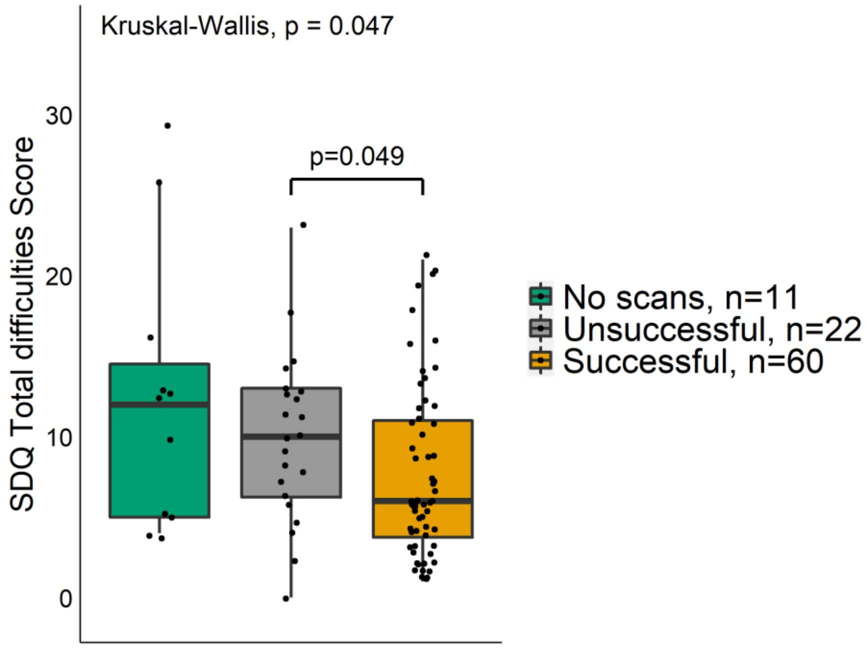

T1w	DWI	T1w_and_DWI	Case_status	MABC_Ma	MABC_Aim	MABC_Bal	MABC_tota
Successful	Successful	Successful	0	6	4	9	6
no scans	no scans	no scans	0	4	14	9	8
Unsuccessful	Unsuccessful	Unsuccessful	0	8	10	16	11
Successful	Successful	Successful	0	7	10	5	6
Successful	Successful	Successful	0	11	10	15	12
Successful	Successful	Successful	0	2	4	8	3
Successful	Successful	Successful	0	5	5	9	6
Unsuccessful	Successful	Unsuccessful	0	5	10	4	5
no scans	no scans	no scans	0	10	13	11	12
Successful	Successful	Successful	0	10	10	11	11
Successful	Successful	Successful	0	5	5	10	6
Successful	Unsuccessful	Unsuccessful	0	12	8	9	10
Successful	Successful	Successful	0	12	11	14	13
Successful	Successful	Successful	0	5	12	10	11
no scans	no scans	no scans	0	7	9	8	7
no scans	no scans	no scans	0	4	7	5	4
Unsuccessful	Successful	Unsuccessful	0	6	9	10	8
Successful	Successful	Successful	0	8	8	4	5
Successful	no scans	Unsuccessful	0	13	8	9	10
Unsuccessful	no scans	Unsuccessful	0	10	8	7	8
Successful	Successful	Successful	1	16	18	12	18
Successful	Successful	Successful	1	9	11	11	10
Unsuccessful	Successful	Unsuccessful	1	9	12	6	8
Successful	Successful	Successful	0	9	8	11	9
Unsuccessful	Successful	Unsuccessful	0	9	9	8	8
Successful	Successful	Successful	1	10	8	6	7
Successful	Successful	Successful	1	8	12	14	11
Successful	Unsuccessful	Unsuccessful	1	12	10	14	12
Successful	Successful	Successful	1	14	5	10	10
Successful	Successful	Successful	1	11	8	11	9
Successful	Successful	Successful	1	3	7	8	5
Successful	Successful	Successful	0	8	9	4	5
no scans	no scans	no scans	0	2	2	1	1
no scans	no scans	no scans	0	15	12	12	15
Successful	Successful	Successful	1	10	15	14	13
Successful	Successful	Successful	1	12	9	11	12
Successful	Successful	Successful	0	1	8	9	4
Successful	Successful	Successful	0	10	7	9	9
Successful	Successful	Successful	1	13	11	14	13
Unsuccessful	Unsuccessful	Unsuccessful	1	12	10	6	9
Successful	Successful	Successful	1	11	12	9	11
Unsuccessful	Successful	Unsuccessful	0	12	9	9	10
Successful	Successful	Successful	1	13	9	11	11
Successful	Successful	Successful	1	9	9	7	7
Successful	Successful	Successful	1	10	9	8	8
Unsuccessful	Successful	Unsuccessful	1	8	9	9	8
Successful	Successful	Successful	1	8	11	14	11
Successful	Unsuccessful	Unsuccessful	1	10	8	7	8
Successful	Successful	Successful	0	18	15	19	19

Successful	Successful	Successful	0	15	10	14	14
Successful	Successful	Successful	0	10	12	15	12
Successful	Successful	Successful	0	14	14	10	14
Successful	Successful	Successful	0	16	12	7	12
Unsuccessful	no scans	Unsuccessful	0	9	18	11	13
Successful	Unsuccessful	Unsuccessful	0	13	15	14	16
Unsuccessful	no scans	Unsuccessful	0	11	9	10	12
Unsuccessful	Unsuccessful	Unsuccessful	0	4	4	5	3
Successful	Successful	Successful	0	5	4	3	3
Successful	Successful	Successful	0	12	12	15	14
Successful	Successful	Successful	0	16	13	14	16
Successful	Successful	Successful	0	14	15	17	17
Successful	Successful	Successful	0	4	7	10	6
no scans	no scans	no scans	1	9	9	8	8
no scans	no scans	no scans	1	12	15	14	15
Successful	Successful	Successful	0	12	9	9	10
no scans	no scans	no scans	0	7	8	11	9
Successful	Successful	Successful	0	8	15	16	13
Unsuccessful	Successful	Unsuccessful	0	8	2	5	4
Successful	Successful	Successful	1	13	15	14	16
Successful	Unsuccessful	Unsuccessful	0	12	10	17	13
Successful	Successful	Successful	1	6	15	14	11
Successful	Successful	Successful	0	12	9	6	8
Successful	Successful	Successful	1	11	8	11	11
Successful	Successful	Successful	1	9	10	11	11
Successful	Successful	Successful	1	18	10	14	15
Successful	Successful	Successful	1	12	7	15	11
Successful	Successful	Successful	1	8	7	13	9
Unsuccessful	Successful	Unsuccessful	1	11	8	11	10
Unsuccessful	Unsuccessful	Unsuccessful	0	9	9	9	9
Successful	Successful	Successful	1	12	9	14	12
Successful	Successful	Successful	1	6	5	12	7
Successful	Successful	Successful	1	13	8	11	11
no scans	no scans	no scans	1	13	10	12	13
Successful	Successful	Successful	1	15	9	14	13
no scans	no scans	no scans	1	6	15	11	11
Successful	Unsuccessful	Unsuccessful	0	12	9	11	11
Successful	Successful	Successful	1	12	9	12	11
Successful	Successful	Successful	1	11	13	11	13
Successful	Successful	Successful	1	11	10	15	12
Successful	Successful	Successful	1	9	8	5	6
Successful	Successful	Successful	1	5	9	9	7
Successful	Successful	Successful	1	15	13	11	15
Successful	Successful	Successful	1	15	13	12	15

SDQ_hyper	SDQ_TotalI	WISC_Verb	WISC_Worl	WISC_Proc	WISC_FSIQ	Sex	Age_montl	Ave_absolu
0	0	83	86	121	86	Male	84	0.66
7	7	93	91	88	85	Female	90	
2	2	99	97	103	99	Female	88	3.47
3	3	100	107	88	97	Female	91	1.69
1	1	98	104	94	103	Female	92	0.79
3	3	73	62	75	62	Male	86	0.68
0	0	108	80	88	88	Female	81	0.73
7	7	81	83	91	81	Female	93	5.67
2	2	95	107	100	99	Female	83	
2	2	100	83	103	92	Male	78	0.6
1	1	95	77	109	85	Male	87	0.89
2	2	89	77	94	88	Female	83	13.92
2	2	108	97	97	99	Male	83	1.51
1	1	112	113	94	106	Female	79	3.32
6	6	93	88	88	86	Female	88	
3	3	99	102	97	89	Female	87	
3	3	93	104	100	92	Male	80	2.31
2	2	98	113	109	103	Female	84	1.42
2	2	108	99	100	99	Male	87	
3	3	95	107	109	103	Female	82	
1	1	106	104	141	125	Male	91	0.91
6	6	89	80	100	87	Female	74	1.03
0	0	102	91	126	103	Female	86	1.39
1	1	89	99	73	81	Female	78	2.09
0	0	93	102	128	107	Male	83	2.87
0	0	116	110	106	119	Female	78	1.63
2	2	104	88	97	95	Female	72	1.14
2	2	108	91	85	96	Female	81	2.31
1	1	114	113	123	119	Female	73	0.73
0	0	104	135	100	114	Female	93	0.77
4	4	93	113	97	99	Male	78	0.66
1	1	85	88	97	89	Female	95	1.06
4	4	85	80		81.3	Female	86	
2	2	93	113	100	96	Female	78	
1	1	87	83	68	75	Female	90	2.09
0	0	102	110	106	104	Female	91	0.67
2	2	98	71	78	77	Male	73	1.12
1	1	89	88	78	84	Female	77	2.33
0	0	121	113	128	127	Female	82	1.88
0	0	104	80	83	91	Male	76	7.69
0	0	81	102	118	97	Female	79	1.32
1	1	89	97	68	81	Male	86	2.18
2	2	104	110	109	106	Female	80	1.51
2	2	110	110	106	114	Male	89	1.63
5	5	102	102	126	103	Female	92	0.8
1	1	95	94	106	99	Female	74	3.17
0	0	100	110	97	108	Male	82	0.62
4	4	114	107	121	120	Male	89	10.34
2	2	108	113	109	114	Female	79	3.01

2	2	96	116	94	99	Female	77	0.83
4	4	96	94	85	89	Male	82	2.17
1	1	108	107	103	103	Male	94	2.27
4	4	126	104	88	105	Female	83	1.92
5	5	89	86	83	84	Female	84	
7	7	96	97	109	102	Female	95	4.85
3	3	110	99	115	106	Female	95	
1	1	98	104	106	102	Male	74	4.53
8	8	108	83	103	93	Male	84	1.26
3	3	96	91	112	90	Female	84	1.02
6	6	95	91	100	89	Male	79	1.49
2	2	110	91	136	114	Male	91	0.95
3	3	116	102	88	105	Female	88	2.73
3	3	106	102	109	111	Male	75	
3	3	100	88	109	96	Male	76	
5	5	85	88	121	92	Female	95	1.72
5	5	81	74	109	86	Male	84	
6	6	116	107	131	114	Male	78	1.34
2	2	99	97	112	103	Male	77	0.84
5	5	112	97	94	108	Female	83	0.79
8	8	83	74	115	84	Male	95	4.12
0	0	104	94	85	97	Female	80	3.42
1	1	112	107	97	115	Female	78	1.14
0	0	114	104	109	119	Female	75	0.52
4	4	110	104	97	108	Female	87	0.68
2	2	114	107	106	108	Male	91	0.89
2	2	110	99	97	111	Male	85	1.65
1	1	124	102	121	120	Male	92	1.51
6	6	119	116	94	119	Female	75	2.33
4	4	93	74	88	88	Male	95	2.66
1	1	114	77	103	99	Male	79	2.78
2	2	108	99	109	110	Male	91	1.24
7	7	110	107	85	111	Female	87	1.11
3	3	134	129	128	137	Female	87	
1	1	106	104	109	108	Male	87	1.32
0	0	116	129	103	121	Female	80	
2	2	121	110	106	123	Male	87	2.81
0	0	119	110	85	114	Male	87	1.97
2	2	121	110	91	111	Male	82	1
6	6	110	126	118	119	Male	87	1.63
10	10	100	104	109	88	Female	82	2.03
2	2	99	123	103	92	Male	73	1.35
4	4	126	113	126	137	Female	90	0.81
5	5	119	110	126	132	Male	94	1

Ave_relative_motion
0.38
1.13
0.54
0.27
0.33
0.29
0.99
0.41
0.28
1.2
0.36
0.83
0.7
0.34
0.36
0.26
0.53
0.39
0.42
0.43
0.34
0.45
0.25
0.31
0.34
0.42
0.89
0.32
0.32
0.86
0.38
0.48
0.34
1.21
0.33
0.75
0.27
0.81
0.46
0.63
0.72

0.55
0.75
0.59
1
0.6
1.02
0.5
0.78
0.52
0.72
1.42
0.86
0.54
0.47
0.5
0.99
0.55
0.49
0.46
0.49
0.38
0.43
0.52
0.92
0.84
0.4
0.41
0.59
0.41
0.38
0.43
0.39
0.38
0.44
0.46
0.42
0.41
