# Supplementary material for: Factors associated with MRI success in children cooled for neonatal encephalopathy and controls
Source: Pediatr Res. 2022 Jul 29;93(4):1017–23. doi: 10.1038/s41390-022-02180-y (PMC10033414; doi:10.1038/s41390-022-02180-y)

Supplementary table 1. Participant demographics for children who underwent MRI scans.

	**Cases (n=43)**	**Controls (n=39)**	***P* value**
Age, median (range)	7.0 (6.0-7.9)	7.1 (6.1-7.9)	0.941
Sex, male/female	21/22	17/22	0.634
Index of Multiple Deprivation, median (range)	7 (1-10)	7 (2-10)	0.407

Supplementary table 2. Summary of developmental outcome scores for children with and without successful MRI scans.

Developmental outcome	Unsuccessful/Successful MRI, mean (SD)	
**Comprehension Skills**		
WISC-IV Verbal comprehension	*Unsuccessful/successful T1w:*<br>*Unsuccessful/successful DWI:*<br>*≥1 unsuccessful/2 successful scans:*	97.27 (9.16)/ 104.18 (11.80)<br>100.50 (11.81)/ 103.41 (11.81)<br>99.00 (10.81)/ 104.35 (11.67)
WISC-IV Working memory	*Unsuccessful/successful T1w:*<br>*Unsuccessful/successful DWI:*<br>*≥1 unsuccessful/2 successful scans:*	95.40 (11.12)/ 99.85 (13.85)<br>91.10 (13.96)/ 100.28 (13.33)<br>94.82 (11.75)/ 100.58 (13.77)
WISC-IV Processing Speed	*Unsuccessful/successful T1w:*<br>*Unsuccessful/successful DWI:*<br>*≥1 unsuccessful/2 successful scans:*	100.80 (16.57)/ 102.93 (15.48)<br>101.00 (12.96)/ 102.81 (16.21)<br>101.91 (15.15)/ 102.77 (15.89)
WISC-IV Full-Scale IQ	*Unsuccessful/successful T1w:*<br>*Unsuccessful/successful DWI:*<br>*≥1 unsuccessful/2 successful scans:*	97.20 (10.78)/ 102.54 (14.42)<br>99.30 (13.23)/ 102.10 (14.31)<br>98.64 (12.06)/ 102.63 (14.49)
**Co-ordination skills**		
MABC-2 Manual Dexterity	*Unsuccessful/successful T1w:*<br>*Unsuccessful/successful DWI:*<br>*≥1 unsuccessful/2 successful scans:*	8.73 (2.37)/ 10.40 (3.74)<br>10.40 (2.76)/ 10.01 (3.78)<br>9.77 (2.54)/ 10.22 (3.90)
MABC-2 Aiming and Catching	*Unsuccessful/successful T1w:*<br>*Unsuccessful/successful DWI:*<br>*≥1 unsuccessful/2 successful scans:*	9.07 (3.47)/ 9.82 (3.08)<br>9.30 (2.73)/ 9.68 (3.14)<br>9.27 (3.15)/ 9.83 (3.16)
MABC-2 Balance	*Unsuccessful/successful T1w:*<br>*Unsuccessful/successful DWI:*<br>*≥1 unsuccessful/2 successful scans:*	8.40 (3.09)/ 11.07 (3.43)<br>10.80 (4.26)/ 10.63 (3.49)<br>9.41 (3.50)/ 11.02 (3.44)
MABC-2 Total score	*Unsuccessful/successful T1w:*<br>*Unsuccessful/successful DWI:*<br>*≥1 unsuccessful/2 successful scans:*	8.40 (2.77)/ 10.57 (3.62)<br>10.20 (3.43)/ 10.10 (3.68)<br>9.36 (3.02)/ 10.43 (3.73)
**Hyperactivity**		

SDQ Hyperactivity	*Unsuccessful/successful T1w:*<br>*Unsuccessful/successful DWI:*<br>*≥1 unsuccessful/2 successful scans:*	2.53 (2.20)/ 2.57 (2.30)<br>3.20 (2.57)/ 2.43 (2.28)<br>2.95 (2.36)/ 2.42 (2.24)
SDQ Total Difficulties	*Unsuccessful/successful T1w:*<br>*Unsuccessful/successful DWI:*<br>*≥1 unsuccessful/2 successful scans:*	9.40 (4.69)/ 7.99 (5.56)<br>9.40 (6.41)/ 7.88 (5.32)<br>9.91 (5.26)/ 7.63 (5.38)

Supplementary figure 1. Example of successful and unsuccessful T1w images and DWIs. (A) shows an example of a successful T1w image, and (B-C) show examples of unsuccessful T1w images due to blurring or ghosting. (D) shows an example of a successful DWI, and (E-F) show examples of unsuccessful DWIs.

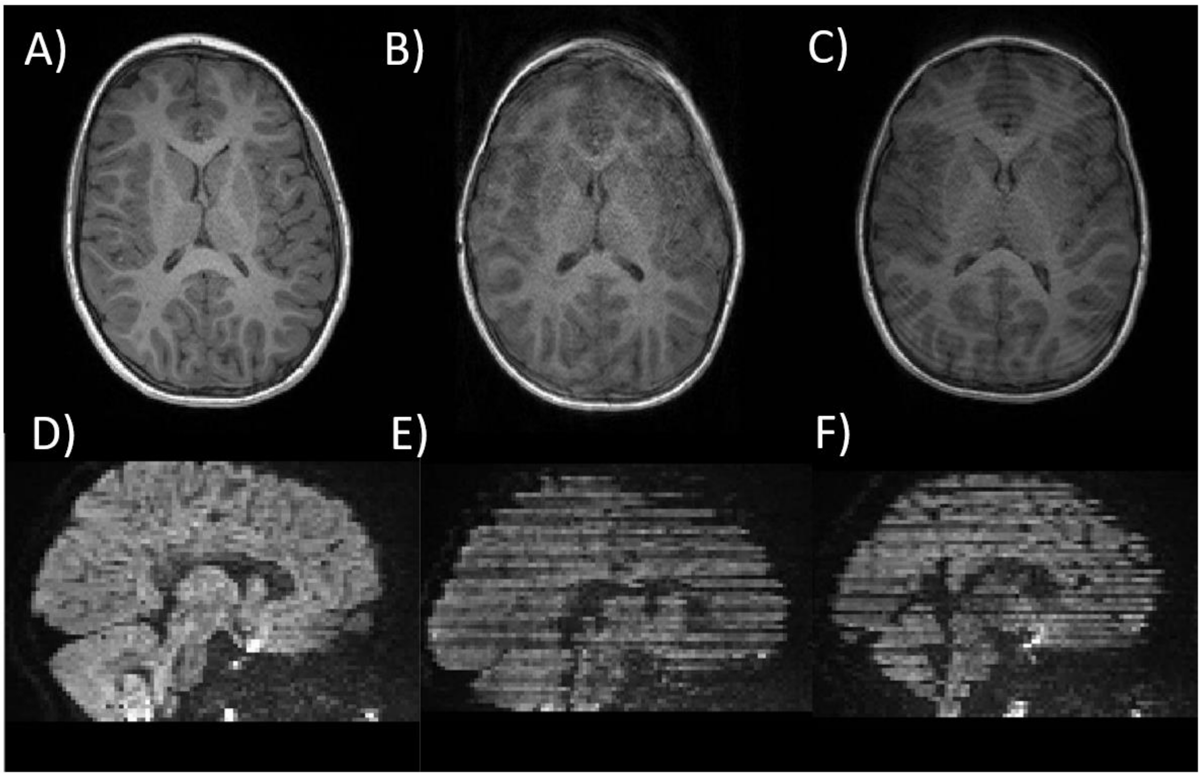

Supplementary figure 2. Comparison of SDQ total difficulties scores between children who did not go in the scanner (n=11), children with at least one unsuccessful MRI (T1w or DWI) (n=22) and children with successful MRIs (n=60).

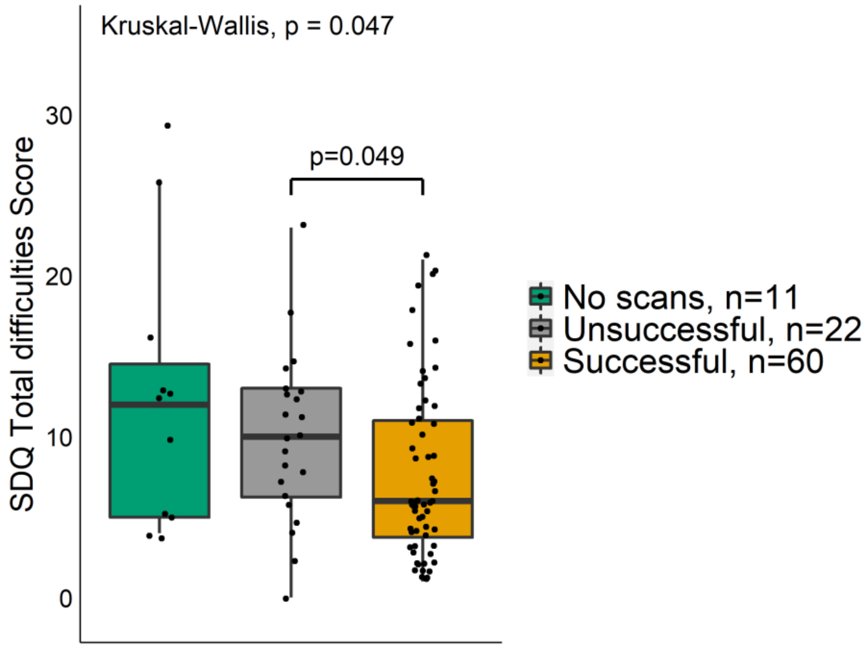

Supplement: Supplementary file 1 [file 41390_2022_2180_MOESM1_ESM.pdf]
